# Emotion Recognition Using Eye-Tracking: Taxonomy, Review and Current Challenges

**DOI:** 10.3390/s20082384

**Published:** 2020-04-22

**Authors:** Jia Zheng Lim, James Mountstephens, Jason Teo

**Affiliations:** 1 Evolutionary Computing Laboratory, Faculty of Computing and Informatics, Universiti Malaysia Sabah, Jalan UMS, Kota Kinabalu 88400, Sabah, Malaysia; takila_111@hotmail.com; 2 Faculty of Computing and Informatics, Universiti Malaysia Sabah, Jalan UMS, Kota Kinabalu 88400, Sabah, Malaysia; james@ums.edu.my

**Keywords:** affective computing, emotion recognition, eye-tracking, machine learning, emotion engineering

## Abstract

The ability to detect users’ emotions for the purpose of emotion engineering is currently one of the main endeavors of machine learning in affective computing. Among the more common approaches to emotion detection are methods that rely on electroencephalography (EEG), facial image processing and speech inflections. Although eye-tracking is fast in becoming one of the most commonly used sensor modalities in affective computing, it is still a relatively new approach for emotion detection, especially when it is used exclusively. In this survey paper, we present a review on emotion recognition using eye-tracking technology, including a brief introductory background on emotion modeling, eye-tracking devices and approaches, emotion stimulation methods, the emotional-relevant features extractable from eye-tracking data, and most importantly, a categorical summary and taxonomy of the current literature which relates to emotion recognition using eye-tracking. This review concludes with a discussion on the current open research problems and prospective future research directions that will be beneficial for expanding the body of knowledge in emotion detection using eye-tracking as the primary sensor modality.

## 1. Introduction

With the development of advanced and affordable wearable sensor technologies, investigations into emotion recognition have become increasingly popular among affective computing researchers since emotion recognition can contribute many useful applications in the fields of neuromarketing, entertainment, computer gaming, health, psychology, and education, among others. Emotions play an important role in human activity and real-life interactions. In recent years, there has been a rising trend in research to improve the emotion recognition systems with the ability to detect, process, and respond to the user’s emotional states. Since emotions contain many nonverbal cues, various studies apply different modalities as indicators of emotional states. There are many applications have been developed with emotion detection such as safe driving, mental health monitoring, and social security [1].

Many studies have focused on the interaction between users and computers. Hence, Human-Computer Interaction (HCI) [2] has become an increasingly important field of computer science research. HCI plays an important role in recognizing, detecting, processing, and respond to the user’s emotions. The studies of Fischer et al. [3] and Cowie et al. [4] focus on user modeling in HCI and emotion recognition in HCI. Computer systems that can detect human emotion are called affective computer systems. Affective computing is the area of study which combines the fields of computer science, psychology, cognitive science as well as artificial intelligence and proposing devices that are capable of recognizing, reading, storing and responding to human emotions. It attempts to gather useful information from human behavior to measure and process human emotions. It has emerged as an important field of study that aims to develop systems that can automatically recognize human emotions. The study of is a review of affective computing. An emotion recognition system will help in detecting human emotions based on the information that is obtained from the various sensors such as eye-tracking and electroencephalography (EEG) data, among others. In the tasks of emotion detection, various signals can be used to classify emotions based on physiological as well as non-physiological signals. Early works relied more on human physical signals such as text, speech, gesture, facial expressions, and posture [5]. Recently, many papers have reported on experiments that were conducted using physiological signals, such as EEG brainwave signals, pupil responses, electrooculography (EOG), electrocardiogram (ECG), electromyogram (EMG), as well as galvanic skin response (GSR). The study of Shu et al. [6] reviewed the papers on emotion recognition based on physiological signals.

Eye-tracking is the process of measuring where and when the user’s eyes are focused, or in other words, the point of gaze, as well as the size of the pupil. An eye-tracker is a device for measuring an individual’s eye positions and eye movements. It is a sensor technology that provides a better understanding of the user’s visual attention. The camera monitors light source reflection along with visible eye features such as the pupil. The eye-tracker also detects additional information such as blink frequency and pupil diameter changes. The seminal work of Hess et al. [7] reported that the increase in the pupil size of the eyes is observed to accompany emotionally toned or fascinating visual stimulus viewing. Additionally, many other features can be utilized to recognize emotions apart from pupil diameter, including fixation duration, saccade, and EOG signals. Eye movement signals are thus widely used in HCI research since they can be used as an indication of the user’s behaviors. Most of the previous studies have used eye movements to analyze the interest of users, visual search processes, and information processing [8]. Hence, eye-tracking has become more popular in the area of cognitive science and affective information processing [9]. There is also prior work reported on the use of eye movement analysis for activity recognition, but not emotion recognition, using EOG signals [10]. 

Eye movement signals allow us to pinpoint what is attracting the user’s attention and observe their subconscious behaviors. They can be important cues for context-aware environments, which contain complementary information for emotion recognition. The signals can provide some emotional-relevant features to determine the emotional states of a user. An investigator can estimate the emotion of the user based on the changes in their pupil size. For example, the pupil diameter is larger when the emotions are in the positive valence, it can be defined that the pupil size will become bigger when an individual has a positive feeling. It will be very helpful since measuring an eye feature need only a simple camera. The raw data of fixation duration allows us to know which scenes of a video presentation are attracting the user’s attention or what is making the user happy or upset. The relevant eye features and the raw data from the stimuli are then used for classification and the results are based on the accuracy of the recognition performance by machine learning algorithms or other classifiers. However, there are only a very limited number of studies that have developed effective features of eye movements for emotion recognition thus far.

**Figure 1:**
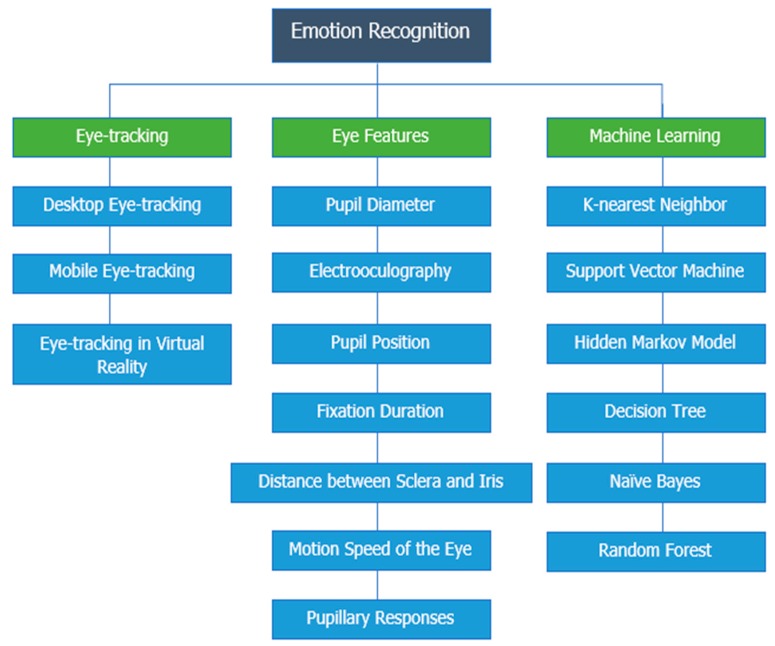
Taxonomy of emotion recognition using eye-tracking.

In this survey paper, we review the studies and works that present the methods for recognizing emotions based on eye-tracking data. Section 2 presents the methodology of this survey paper. Section 3 describes the brief introduction and background of emotions and eye-tracking. The features extracted from eye-tracking data with the emotional-relevant features are presented in Section 4. These features include pupil diameter, EOG signals, pupil position, fixation duration, the distance between sclera and iris, motion speed of the eye, and pupillary responses. Section 5 presents the critical summary of this paper with a table of comparison of previous studies and investigations. The final section discusses the current open problems in this research domain and possible avenues for future work that could benefit this field of research. A taxonomy of this paper is shown in Figure 1.

## 2. Background

In this section, a brief introduction to human emotions and eye-tracking will be presented which includes the various emotion models, emotion stimulation tools, and eye-tracking approaches commonly adopted in desktop setups, mobile devices, as well as in virtual reality headsets.

### 2.1. Human Emotions

Emotions are a mental state that are experienced by a human and is associated with feelings and a degree of pleasure or displeasure [11]. Emotion is often intertwined with mood, temperament, personality, disposition, and motivation. They can be defined as a positive (pleasure) or negative (displeasure) experience from different physiological activities. They are states of feelings that result in psychological changes that influence human actions or behavior [12]. Emotions are complex psychological states that contain different components, such as subjective experience, psychophysiological responses, behavioral expressive responses, and cognitive processes [13]. In Scherer’s components, there are five crucial elements of emotion which are cognitive appraisal, bodily symptoms, action tendencies, expression, and feelings [14]. Emotions have been described as responses to major internal and external events. Emotions are very important but difficult to quantify and agreed on since different researchers use different and often incompatible definitions and emotional ontologies. This makes emotion research often a very challenging area to work in since the comparison between studies is not always appropriate.

Classification of emotions is normally approached through categorizing emotions as being discrete in nature. In discrete emotion theory, all humans have an inborn set of basic emotions that can be recognized cross-culturally. These basic emotions are said to be discrete because they are distinguishable by an individual’s countenance and biological processes [15]. Ekman’s model proposes that emotions are indeed discrete and suggests that these emotions can be universally recognized. Ekman classifies six basic emotions from his research findings, which are anger, disgust, fear, happiness, sadness, and surprise [16]. The list of these emotions is then extended and classified into both facial and vocal expressions. Plutchik’s model proposes eight basic emotions: joy, sadness, anger, fear, trust, disgust, surprise, and anticipation [17]. The wheel of emotions is thus developed where these eight basic emotions are grouped to either being of a positive or negative nature.

**Figure 2:**
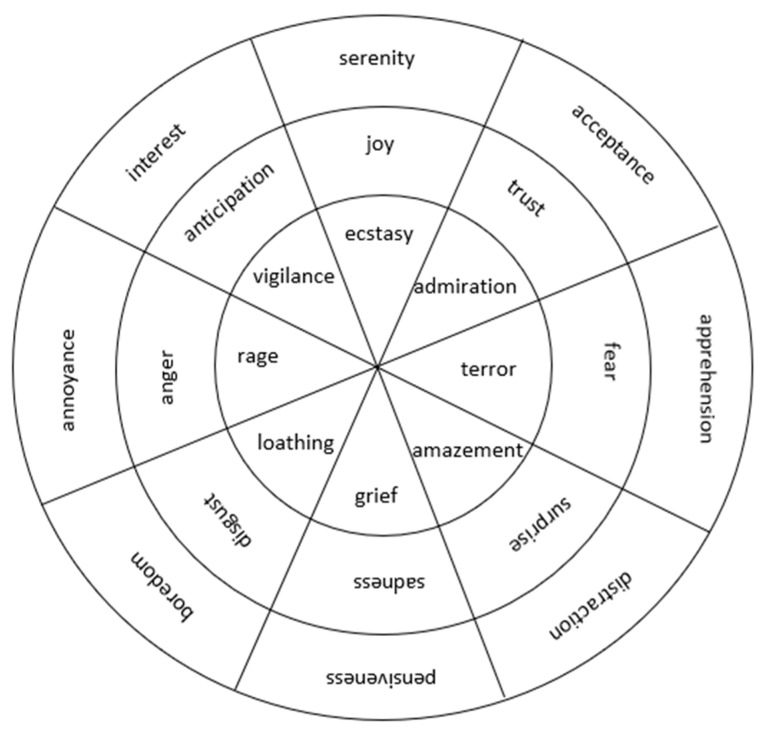
Wheel of emotions.

Emotion classifications and the closely related field of sentiment analysis can be conducted through both supervised and unsupervised machine learning methodologies. The most famous usage of this analysis is the detection of sentiment on Twitter. In recent work, Mohammed et al. proposed an automatic system called a Binary Neural Network (BNet) to classify multi-label emotions by using deep learning for Twitter feeds [18]. They conducted their work on emotion analysis with the co-existence of multiple emotion labels in a single instance. Most of the previous work only focused on single-label classification. A high-level representation in tweets is first extracted and later modeled using relationships between the labels that correspond to the eight emotions in Plutchik’s model (joy, sadness, anger, fear, trust, disgust, surprise, and anticipation) and three additional emotions of optimism, pessimism, and love. The wheel of emotions by Plutchik describes these eight basic emotions and the different ways they respond to each other, including which ones are opposites and which ones can easily convert into one another (Figure 2). 

**Figure 3:**
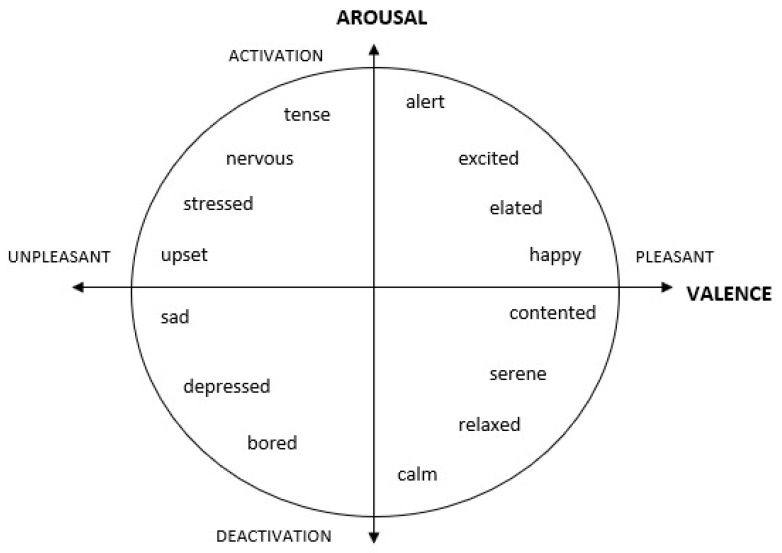
A graphical representation of the Circumplex Model of Affect.

Arguably, the most widely used model for classifying human emotions is known as the Circumplex Model of Affects (Figure 3), which was proposed by Russell et al. [19]. It is distributed in a two-dimensional circular space comprising the axes of arousal (activation/deactivation) and valence (pleasant/unpleasant). Each emotion is the consequence of a linear combination of these two dimensions, or as varying degrees of both valence and arousal. Valence represents the horizontal axis and arousal represents the vertical axis, while the circular center represents a neutral level of valence and arousal [20]. There are four quadrants in this model by combining a positive/negative valence and a high/low arousal. Each of the quadrants represents the respective emotions. The interrelationships of the two-dimensional combination are represented by a spatial model. Quadrant 1 represents happy/excited emotions which are located at the combination of high arousal and positive valence; quadrant 2 represents stressed/upset emotions which are located at the combination of high arousal and negative valence; quadrant 3 represents sad/bored emotions which are located at the combination of low arousal and negative valence, and quadrant 4 represents calm/relaxed emotions which are located at the combination of low arousal and positive valence.

### 2.2. Emotion Stimulation Tools

There are many ways that can be used to stimulate an individual’s emotions such as through watching a movie, listening to a piece of music, or simply looking at a still image. Watching a movie, for example, could potentially evoke various emotional states due to different responses evoked from watching different segments or scenes in the movie. In the work of Soleymani et al. [21], the authors used EEG, pupillary response, and gaze distance to get the responses of users from video clips. 30 participants started with a short neutral video clip, then one of the 20 video clips are played randomly from the dataset. EEG and gaze data are recorded and extracted based on the participant’s responses where three classes each for both arousal and valence were defined (calm, medium aroused, and activated; unpleasant, neutral, and pleasant). Another study on emotion recognition utilized heartbeats to evaluate human emotions. In the work of Choi et al. [22], the emotion stimulation tool used by the authors is International Affective Picture System (IAPS), which was proposed by Lang et al. [23]. The selected photographs were displayed randomly to participants for 6 s for each photograph with 5 s rest before beginning the viewing and 15 s after a photograph was shown. A Self-Assessment Manikin (SAM) was used to analyze the happy (positive) emotion and unhappy (negative) emotion.

### 2.3. Eye-Tracking

Eye-tracking is the process of determining the point of gaze or the point where the user is looking at for a particular visual stimulus. An eye-tracker is a device for eye-tracking to measure an individual’s eye positions and eye movements [24]. The acquisition of the eye-tracking data can be conducted in several ways. Essentially, there are three eye-tracker types, which are eye-attached tracking, optical tracking, and electric potential measurement. Currently, eye-tracking technology has been applied to many areas including cognitive science, medical research, and human-computer interaction. Eye-tracking as a sensor technology that can be used in various setups and applications is presented by Singh et al. [25] while another study presents the possibility of using eye movements as an indicator of emotional recognition [26]. There are also studies that describe how eye movements and their analysis can be utilized to recognize human behaviors [27,28]. Numerous researches and studies on eye-tracking technology have been published and the number of papers has steadily risen in recent years.

#### 2.3.1. Desktop Eye-Tracking

**Figure 4:**
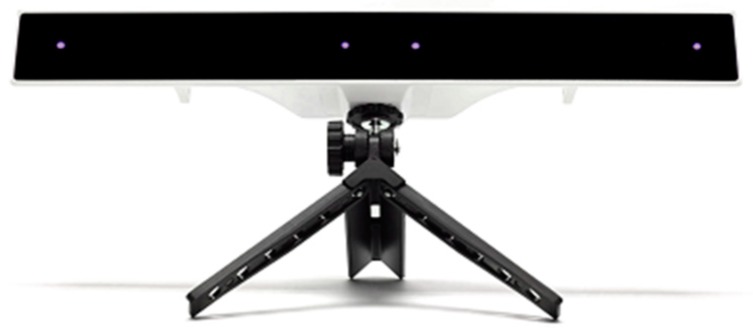
Gazepoint GP3 eye-tracker.

A desktop computer that comes with an eye-tracker can know what is attracting the user’s attention. High-end desktop eye-trackers typically utilize infrared technology as their tracking approach. One such eye-tracker, called the Tobii 4C (Tobii, Stockholm, Sweden), consists of cameras, projectors, and its accompanying image-processing algorithms. Tobii introduced eye-tracking technology to PC gaming in an effort to improve gameplay experiences and performance when the gamers are positioned in front of their computer screens. Another similar device is the GP3 desktop eye-tracker (Figure 4) from Gazepoint (Vancouver, Canada) which is accompanied by their eye-tracking analysis software, called Gazepoint Analysis Standard software. Desktop eye-tracking can also be conducted using low-cost webcams that commonly come equipped on practically all modern laptops. Most of the open-source software for processing eye-tracking data obtained from such low-cost webcams is typically straightforward to install and use, although most have little to no technical support. Furthermore, webcam-based eye-tracking is much less accurate compared to infrared eye-trackers. Moreover, webcam-based eye-tracking will not work well or at all in low light environments.

#### 2.3.2. Mobile Eye-Tracking

**Figure 5:**
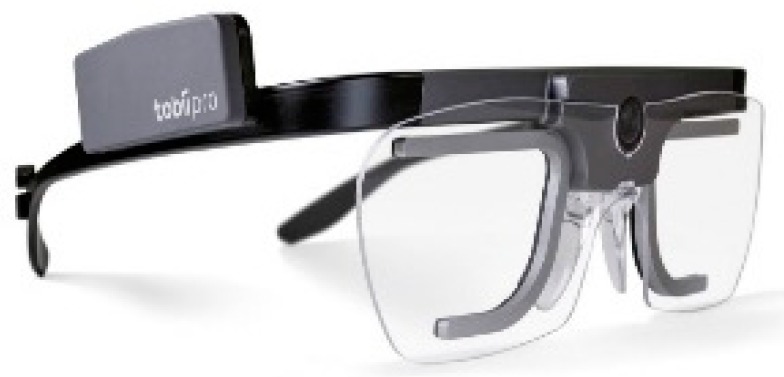
Tobii Pro Glasses 2 eye-tracker.

A mobile eye-tracker is typically mounted onto a lightweight pair of glasses. It allows the user to move freely in their natural environment and at the same time captures their viewing behavior. Mobile eye-trackers can also be used for marketing purposes and manufacturing environments, for example in measuring the cognitive workload of forklift drivers. It is easy to use and the eye-tracking data is captured and recorded in the application of a mobile phone. The user can view the data on their phone with the connected wearable eye-tracker via Bluetooth. The Tobii Pro Glasses 2 product (Figure 5) is also currently available in the market. Researchers may begin to understand the nature of the decision-making process by studying how visual activity is eventually related to people’s actions in different situations. It is possible to process the mobile eye-tracking (MET) data as many times as needed without requiring the need to do repeated testing. MET also takes us much closer to the consumer’s mind and feelings. They can capture the attention of consumers and know what their customers are looking for and what they care about. The cons of MET are that they must be used in highly controlled environments. MET is also rather costly for typical everyday consumers who may want to use it. 

#### 2.3.3. Eye-Tracking in Virtual Reality

**Figure 6:**
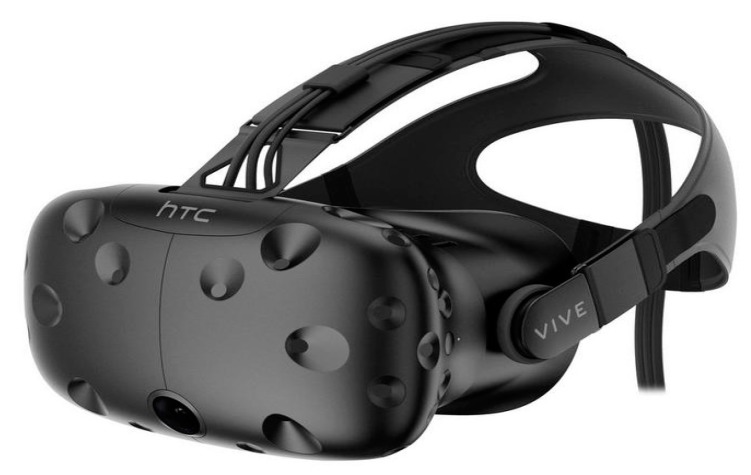
HTC Vive VR headset.

**Figure 7:**
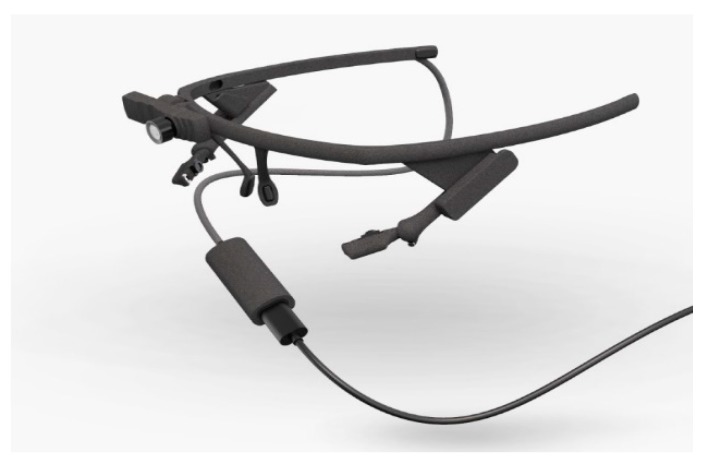
Pupil Labseye-tracker.

**Figure 8:**
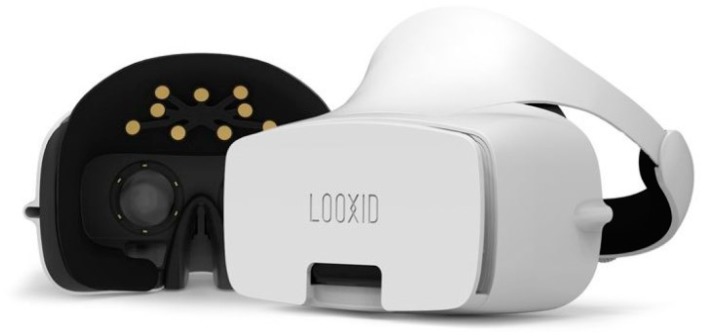
LooxidVR headset.

Many virtual reality (VR) headsets are now beginning to incorporate eye-tracking technology into their head-mounted display (HMD). The eye-tracker works as a sensor technology that provides a better understanding of the user’s visual attention in VR. VR may create any type of virtual environment for its users while eye-tracking gives insights into where the user’s visual attention is at for each moment of the experience. As such, eye movement signals can be used to provide a natural and efficient way to observe the behaviors of VR users and allow them to find out what is attracting a user’s attention in the VR’s simulated environment. Some VR headsets may not have a built-in eye-tracker, for example, the HTC Vive (HTC, Taipei, Taiwan, Figure 6). There is however the possibility of adding on a third-party eye-tracker into the headset, such as the eye-tracker produced by Pupil Labs (Berlin, Germany, Figure 7), which has a very thin and extremely lightweight design and profile. The VR-ready headset Looxid VR [29] produced by Looxid Labs (Daejeon, South Korea) integrates an HMD with built-in EEG sensors and eye-tracking sensors in addition to a slot for inserting a mobile phone to display VR content (Figure 8). This approach allows for the straightforward synchronization and simultaneous acquisition of eye-tracking and matching EEG data resulting in high fidelity synchronized eye-tracking plus EEG data for VR experiences. However, the main drawbacks of both the Pupil Labs and Looxid eye-tracking solutions for VR are that they are very costly for the everyday consumer.

## 3. Emotional-Relevant Features from Eye-tracking

This section will present the investigations that have been reported in the literature for the extraction of useful features from eye-tracking data for emotion classification. As an example, in the study of Mala et al. [30], the authors report on the use of optimization techniques for feature selection based on a differential evolution algorithm in an attempt to maximize the emotional recognition rates. Differential evolution is a process that optimizes the solution by iteratively attempting to improve the candidate solution for a given quality measure and it keeps the best score for the solution. In this section, the emotional-relevant features will be discussed including pupil diameter, EOG signals, pupil position, fixation duration, the distance between sclera and iris, motion speed of the eye, and pupillary responses.

### 3.1. Pupil Diameter

In the work of Lu et al. [31], the authors combine the eye movements with EEG signals to improve the performance of emotion recognition. The work showed that the accuracy of combining eye movements and EEG is higher than the accuracies of solely using eye movements data only and using EEG data only respectively. Power spectral density (PSD) and differential entropy (DE) were extracted from EEG signals. STFT was used to compute the PSD in five frequency bands: delta (1 to 4 Hz), theta (4 to 8 Hz), alpha (8 to 14 Hz), beta (14 to 31 Hz), and gamma (31 to 50 Hz) [32] while the pupil diameter is chosen as the eye-tracking feature. PSD and DE features were computed in X and Y axes in four frequency bands: (0–0.2 Hz, 0.2–0.4 Hz, 0.4–0.6 Hz, and 0.6–1.0 Hz) [21]. The eye movement parameters included pupil diameter, dispersion, fixation duration, blink duration, saccade, and event statistics such as blink frequency, fixation frequency, fixation duration maximum, fixation dispersion total, fixation dispersion maximum, saccade frequency, saccade duration average, saccade amplitude average, and saccade latency average. The classifier used is Fuzzy Integral Fusion Strategy. It used a fuzzy measure concept. Fuzzy measure considers simplified measures that replacing the additive property with the weaker monotonicity property. The highest accuracy obtained is 87.59%, while the accuracy of eye movements and EEG alone is 77.80% and 78.51% respectively.

In the work of Partala et al. [33], the authors used auditory emotional stimulation to investigate the pupil size variation. The stimulation was carried out by using International Affective Digitized Sounds (IADS) [34]. The PsyScope program was used to control the stimulation [35]. The results were measured on two dimensions: valence and arousal.

In the study of Oliva et al. [36], the authors explored the relationship between pupil diameter fluctuations and emotion detection by using nonverbal vocalization stimuli. They found that the changes in pupil size were correlated to cognitive processing [37]. It is projected that the changes in baseline pupil size correlated with task efficiency. Increases in pupil diameter associated with task disengagement while decreases in pupil diameter correlated with task participation. They aimed to test stimuli varied in valence, intensity, duration, and ease of identification. Thirty-three university students were chosen as subjects within the age range of 21 to 35. The experiment was carried out using visual and auditory stimuli through the use of Psychopy [38], which consisted of 72 sounds. The neutral emotional sounds were obtained from the Montreal Affective Voices (MAV) [39]. MAV consists of 90 nonverbal affective bursts that correspond to the eight basic emotions of anger, pain, disgust, sadness, fear, surprise, happiness, and pleasure. A stimulation began after the participant completed the practice trials with positive, negative, and neutral sounds. The Generalized Additive Model (GAM) [40] was applied to the valence of the stimulus. The emotion processing was done with LC-NE (locus coeruleus – norepinephrine) system [41]. The accuracy that achieved in this study was 59%.

The study of Zheng et al. [42] presented an emotion recognition method by combining EEG and eye-tracking data. The experiment was carried out in two parts: first was to recognize emotions with a single feature from EEG signals and eye-tracking data separately; the second was to conduct classification based on decision level fusion (DLF) and feature level fusion (FLF). The authors used film clips as the stimuli and each emotional video clips were around 4 min. Five subjects took part in this test. The ESI NeuroScan system was used to record the EEG signals while the eye-tracking data was collected by using the SMI eye-tracker. Pupil diameter was chosen as the feature for emotion detection and four features were extracted from EEG such as differential asymmetry (DASM), differential entropy (DE), power spectral density (PSD), and rational asymmetry (RASM). The classification was done using the SVM classifier. The results showed that the accuracy of classification with the combination of EEG signals and eye-tracking data is better than single modality. The best accuracies achieved 73.59% for FLF and 72.89% for DLF.

In Lanatà et al. [43], the authors proposed a new wearable and wireless eye-tracker, called Eye Gaze Tracker (EGT) to distinguish emotional states stimulated through images using a head-mounted eye-tracking system named HATCAM (proprietary). The stimuli used were obtained from the IAPS set of images. Video-OculoGraphy (VOG) [44] was used to capture the eye’s ambient reflected light. They used Discrete Cosine Transform (DCT) [45] based on the Retinex theory developed by Land et al. [46] for photometric normalization. The mapping of the eye position was carried out after the implementation of Ellipse fitting [47]. Recurrence Quantification Analysis (RQA) was used for the feature extraction process. The features extracted included the fixation time and pupil area detection. The K-Nearest Neighbor (KNN) algorithm [48] was used as a classifier for pattern recognition. Performance evaluation of the classification task performance was subsequently conducted through the use of a confusion matrix [49].

### 3.2. Electrooculography (EOG)

Electrooculography (EOG) is a method used to measure the corneo-retinal standing potential between the human eye’s forehead and back. The EOG signals are generated by this measurement which brings the voltage drop and electrodes detection. The main uses are in the treatment of ophthalmology and in eye movement analysis. Eye-related features such as EOG that are commonly utilized in e-healthcare systems [50,51,52] have also been investigated for emotion classification. In Wang et al. [53], the authors proposed an automatic emotion perception system using the eye movement information-based algorithm to detect the emotional states of adolescences. They discovered two fusion strategies to improve the performance of emotion perception. These were feature level fusion (FLF) and decision level fusion (DLF). Time and frequency features such as saccade, fixation, and pupil diameter were extracted from the collected EOG signals with six Ag-AgCl electrodes and eye movement videos by using Short-Time Fourier Transform (STFT) to process and transform the raw eye movement data. SVM was used as the method to distinguish between three emotions, which were positive, neutral, and negative. 

In Paul et al. [54], the authors used the audio-visual stimulus to recognize emotion using EOG signals with the Hjorth parameter and a time-frequency domain feature extraction method, which was the Discrete Wavelet Transform (DWT) [55]. They used two classifiers in their study to obtain the classification which was SVM and Naïve Bayes (NB) with Hjorth [56]. Eight subjects consisting of four males and four females took part in this study. The age group range was between 23 to 25. 3 sets of the emotional visual clips were prepared and the duration for each clip was 210 s. The video commenced after 10 s of resting period and there was no rest time between the three video clips. Both horizontal and vertical eye movement data were recorded and the classification rates were determined separately. In both horizontal and vertical eye movements, positive emotions achieved 78.43% and 77.11% respectively, which was the highest accuracy compared to the negative and neutral emotions. 

### 3.3. Pupil Position

**Figure 9:**
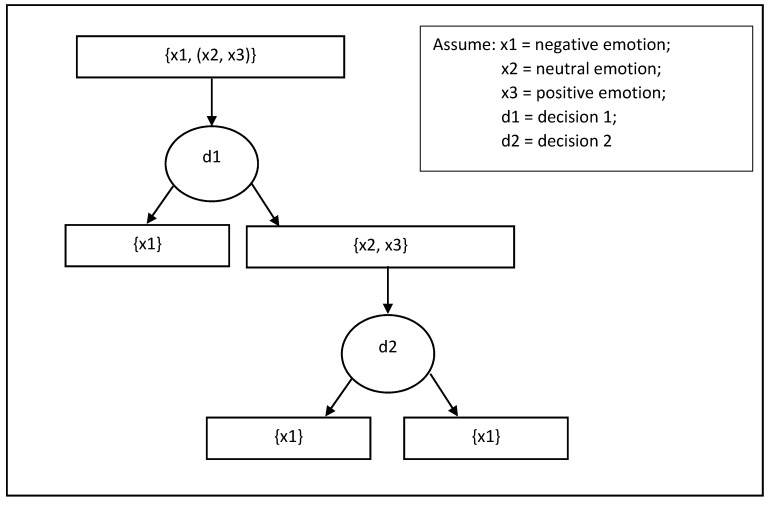
Demonstration of binary decision tree approach.

Aracena et al. [57] used pupil size and pupil position information to recognize emotions while the users were viewing images. The images again were obtained from IAPS and relied on the autonomic nervous system (ANS) response [58] as an indication of the pupil size variation with regards to the image emotional stimulation. Only four subjects covering an age range of 19 to 27 were involved in this experiment. Ninety images were collected randomly from the IAPS dataset in three emotional categories (positive, neutral, negative). The image was presented with a software called the Experiment Builder (SR Research, Ottawa, Canada) in random order for 4 s. Both left and right eyes were recorded at a rate of 500Hz by using the EyeLink 1000 eye-tracker (SR Research, Ottawa, Canada). The pre-processing procedure included blink extraction, saccade extraction, high-frequency extraction, and normalization. The outcomes were measured based on three values of positive, neutral, and negative valence. Finally, they used neural networks (NNs) [59] and binary decision tree (Figure 9) for the classification tasks. The neural network was implemented using Matlab (Mathworks, Natick, MA, USA) via the DeepLearnToolbox [60]. The highest recognition rate achieved was 82.8% and the average of the accuracy was 71.7%.

Recently, a real-time facial expression recognition and eye gaze estimation system was proposed by Anwar et al. [61]. The proposed system can recognize seven emotions: happiness, anger, sadness, neutral, surprise, disgust, and fear. The emotion recognition part was conducted using the Active Shape Model (ASM) developed by Cootes et al. [62] while SVM was used as the classifier for this system. The eye gaze estimation was obtained using the Pose from Orthography and Scaling with Iterations (POSIT) and Active Appearance Model (AAM) [63]. The eye-tracking captured the position and size of the eyes. The proposed system achieved a 93% accuracy.

In Gomez-Ibañez et al. [64], the authors studied the research on facial identity recognition (FIR) and facial emotion recognition (FER) specifically in patients with mesial temporal lobe epilepsy (MTLE) and idiopathic generalized (IGE). The study of Meletti et al. [65] involved impaired FER in early-onset right MTLE. There are also several studies and researches relating to FER and eye movements [66,67,68,69]. These studies suggest that eye movement information can provide important data that can assist in recognizing human emotional states [70,71]. The stimuli of FIR and FER tasks used Benton Facial Recognition Test (BFRT) [72]. The eye movements and fixations were recorded by a high-speed eye-tracking system called the iViewX™ Hi-Speed monocular eye-tracker (Gaze Intelligence, Paris, France) which performed at 1000 Hz. The eye-related features extracted included the number of fixations, fixation time, total duration, and time of viewing. The accuracy of FIR achieved 78% for the control group, 70.7% for IGE, and 67.4% for MTLE. For FER, the accuracy in the control group was 82.7%, 74.3% for IGE, and 73.4% for MTLE.

### 3.4. Fixation Duration

In Tsang et al. [73], the author carried out eye-tracking experiments for facial emotion recognition in individuals with high-functioning autism spectrum disorders (ASD). The participants were seated in front of a computer with a prepared photo on the screen. The eye movements of the participant were recorded by a remote eye-tracker. There was no time limit for every view for each photograph but the next photo was presented if there was no response after 15 s. The gaze behaviors that were acquired included fixation duration, fixation gaze points, and the scan path patterns of visual attention. These features are recorded for further analysis using the areas of interest (AOIs). For the facial emotion recognition (FER) test, analysis of variance (ANOVA) was used to measure the ratings of emotion orientation and emotional intensity. The accuracy achieved 85.48%. While in Bal et al. [74], the authors also work on emotion recognition with ASD but specifically in children only. They classified the emotions by evaluating the Respiratory Sinus Arrhythmia (RSA) [75], heart rate, and eye gaze. RSA is often used for clinical and medical studies [76,77,78]. The emotional expressions were presented using Dynamic Affect Recognition Evaluation [79] system. The ECG recordings [80] and the skeletal muscles’ electrical activity, EMG [81] were collected before the participant started to watch the video stimuli. Three sets of videos were presented randomly. The baseline heart period data were recorded before and after two minutes of the videos being displayed. Emotion recognition included anger, disgust, fear, happiness, surprise, and sadness. In the report by Boraston et al. [82], the potential of eye-tracking technology was investigated for studying ASD. A facial display system called FACE was proposed in the work of Pioggia et al. [83] to verify that this system can help children with autism in developing social skills. 

In Lischke et al. [84], the authors used intranasal oxytocin to improve the emotion recognition of facial expressions. Neuropeptide oxytocin plays a role in the regulation of human emotional, cognitive, and social behaviors [85]. This investigation reported that neuropeptide oxytocin would generally stimulate emotion recognition from dynamic facial expressions and improve visual attention with regards to the emotional stimuli. The classification was done by using Statistical Package for the Social Sciences version 156 (SPSS 15, IBM, Armonk, New York, NY, USA) and the accuracy achieved was larger than 79%. 

### 3.5. Distance Between Sclera and Iris

In Rajakumari et al. [86], the authors recognized six basic emotions in their works namely anger, fear, happiness, focus, sleep, and disgust by using a Hidden Markov Model (HMM), which is widely used machine learning approach [87,88,89,90,91]. The study of Ulutas et al. [92] and Chuk et al. [93] presented the applications of HMM to eye-tracking data. They carried out the studies by measuring the distance between sclera and iris which were then used as features to classify the above mentioned six emotions. 

### 3.6. Eye Motion Speed 

In Raudonis et al. [94], the authors proposed an emotion recognition system that uses eye motion analysis via artificial neural networks (ANNs) [95]. This paper classified four emotions, which were neutral, disgust, amused, and interested. The implementation of the ANN consisted of eight neurons at the input layer, three neurons at the hidden layer, and one neuron for the output layer. In this experiment, three features were extracted, namely the speed of eye motion, pupil size, and pupil position. Thirty subjects were presented with a PowerPoint (Microsoft, Redmond, Washington, DC, USA) slideshow which consisted of various emotional photographs. The average best accuracy of recognition achieved was around 90% and also the highest accuracy obtained was for the classification of the amused emotion. 

### 3.7. Pupillary Responses

Alhargan et al. [96] presented affect recognition by using the pupillary responses in an interactive gaming environment. The features extracted include four frequency bands of PSD features and they are extracted using the STFT. Game researchers reported that emotion recognition can indeed make the game experience richer and improve the overall gaming experience using an affective gaming system [97]. The studies of Zeng et al. [98] and Rani et al. [99] focused on affect recognition using behavioral signals and physiological signals. For the subject in Alhargan’s experiment, it included fourteen students in a range of 26 to 35 with two years or more of gaming experience [96]. Five sets of affective games with different affective labels were used to evoke the responses of the player. The eye movement data of the player were recorded at 250 Hz using Eye-Link II (SR Research, Ottawa, Canada). The experiment commenced by using a neutral game and the next affective game mode was selected randomly. Each player was provided with a SAM questionnaire to rate their experience after playing the games. Pupillary responses were collected by isolating the pupil light reflex (PLR) [100] to extract the useful affective data. SVM was used as the classifier for this work and the Fisher discriminant ratio (FDR) was applied for a good differentiation across the classification. The recognition performance was improved by applying a Hilbert transform to the pupillary response features compared to the emotion recognition without the transform. The accuracy achieved 76% for arousal and 61.4% for valence. In Alhargan et al. [101], another work from the same authors presented a multimodal affect recognition system by using the combination of eye-tracking data and speech signals in a gaming environment. They used pupillary responses, fixation duration, saccade, blink-related measures, and speech signals for recognition tasks. Speech features were extracted by using a silence detection and removal algorithm [102]. The affect elicitation analysis was carried out by using a gaming experience rating feedback from players, as well as eye-tracking features and speech features. It achieved a classification accuracy of 89% for arousal and 75% for valence.

## 4. Summary

In this paper, we have presented a survey on emotion recognition using eye-tracking, focusing on the emotional-relevant features of the eye-tracking data. Several elements relevant to the emotion classification task are summarized, including what emotional stimuli were in the experiment, how many subjects were involved, what emotions were recognized and classified, what features and classifiers were chosen, as well as the prediction rates. Here we present a summary of our main findings from the survey.

From the 11 studies that directly used eye-tracking approaches for the task of classifying emotions, the highest accuracy obtained was 90% using ANN as the classifier with pupil size, pupil position, and motion speed of the eye as the features [94]. Similar to the best outcome of Raudonis et al. [94], it also appears that a combination of training features is required to achieve good classification outcomes as studies that report high accuracies of at least above 85% used at least three features in combination [31,53,73]. The least successful approaches utilized only pupil diameter achieving highly similar and low accuraries of 58.9% [42] and 59.0% [36], respectively. The most commonly used feature (eight studies) was pupil diameter [31,36,42,57,86,94,96,101], followed by fixation duration employed in four studies [31,73,84,101], and finally the least used features were pupil position [57,94] and EOG [53,54], which were used in only two studies each, respectively. The speed of the emotion recognition task was only reported in one of the studies, which could provide classification results within 2 s (with 10% variation) of the presentation of the emotional stimuli [94].

## 5. Directions

The purpose of this paper was to review the investigations related to emotion recognition using eye-tracking. Studies reviewed commenced from papers published starting in 2005 to the most current in 2020 and what was found was that there is only a limited number of investigations that have been reported on emotion recognition using eye-tracking technology. Next, we present a critical commentary as a result of this survey and propose some future avenues of research that will likely be of benefit to further the body of knowledge in this research endeavor. 

### 5.1. Stimulus of the Experiment

There are many methods that can be used to evoke a user’s emotion such as music, video clips, movies, and still images. As we can see from the summary table, images and video clips are most commonly used for the stimulation of the experiment. Most of the images were obtained from the IAPS dataset. However compared to these still images, stimulation in virtual reality (VR) is arguably a more vivid experience since the user can stimulated in an immersive virtual environment. Currently, there is no research that reports on detecting specific emotions in virtual reality using eye-tracking technology. Numerous researches have been conducted for the classification of emotions using different equipment such as EEG. However, there has never been any research being conducted purely on classifying human emotional states by using eye-tracking alone in virtual reality. Although many studies have reported successful emotion recognition, these are purely in non-virtual environments. One of the advantages is that within a VR scene, researchers can stimulate complicated real-life situations to evaluate the complex human behaviors in a fully controllable and mapped environment.

Moreover, the outcomes from emotion recognition studies could sometimes not be entirely accurate since by using images and video clips that are presented by sitting in front of the computer display, such a setup cannot guarantee that the test subject is actually and exactly focusing on the images or the stimulus. In such a setup, the tester’s eyes may be attracted by objects or stimuli apart from that being presented on the computer display, for example, a poster on the wall, a potted plant on the table, or something that makes the test subject lose their focus on the actual stimulus being presented. Some test subjects who are very sensitive to external sound will also most likely lose their attention when a sudden sound made by the surrounding environment stimulates their responses. One way to overcome these limitations of presenting the stimulus via a desktop-based display setup is to make use of a virtual reality stimulus presentation system. Within the VR simulation, the tester will be fully “engulfed” within the immersive virtual environment as soon as they start wearing the VR headset with the integrated earphone. The test subject only can be stimulated by the objects or stimuli within VR scenes and no longer by the surrounding external environment. 

### 5.2. Recognition of Complex Emotions

Many of the studies were classifying positive, negative, and neutral emotions but not a specific emotional state such as happiness, excitement, sadness, surprise, boredom, or disgust. Some studies were only focusing on valence and arousal levels. From the Circumplex Model of Affect, there are four quadrants in this model by combining a positive/negative valence and a high/low arousal. Each of the quadrants represents the respective emotions. One quadrant usually consists of several types of emotions. Some of the studies attempt to classify the happy emotion in quadrant 1 but ignores the fact that alertness, excitement, and elated emotions are also contained within this quadrant. Hence, future works should attempt to further improve the discrimination between such emotions within the quadrant to identify a very specific emotion. For example, we should be able to distinguish between happy and excited emotions since both of them are in quadrant 1 but they are two different emotions. Additionally, more effort should also be put into attempting the recognition of more complex emotions beyond the common six or eight emotions generally reported in emotion classification studies.

### 5.3. The Most Relevant Eye Features for Classification of Emotions

From the limited number of eye-tracking-based emotion recognition studies, a wide variety of features were used to classify the emotions such as the pupillary responses, EOG, pupil diameter, pupil position, fixation time of the eyes, saccades, and motion speed of the eyes. From the survey, there is no clear indication as to which eye feature or a combination of these features is most beneficial for the emotion recognition task. Therefore, a comprehensive and systematic test should be attempted to clearly distinguish between the effectiveness of these various emotional-relevant eye-tracking features for the emotion recognition task.

### 5.4. The Usage of Classifier

There are many classifiers that can be used in emotion classification such as Naïve Bayes, k-nearest neighbor (KNN), decision trees, neural networks, and support vector machines (SVM). A Naïve Bayes classifier applies Bayes Theorem to features of the dataset in a probabilistic manner using the strong and naïve assumption that every feature being classified is independent of the value of any other feature; a KNN classifier conducts its classification using lazy learning and is non-parametric where the training instances are grouped into classes according to the distances to their neighboring distances in order to classify an unseen instance; decision trees represents a branching structure which separates training instances according to rules that are applied to the features of the dataset and classifies new data based on this branching of rules; neural networks are simple computational analogs of synaptic connections in the human brain which accomplishes its learning through adjusting weights of connections between the feature, transformation and output layers of the computational nodes; and SVMs perform classification by attempting to find hyperplanes that most optimally separate between the different classes of the dataset by projecting the dataset into higher dimensions. In classifier analysis, the most important performance metric is accuracy, which is the number of true positive and true negative instances predicted divided by the total number of instances. Most of the studies chosen SVM as their classifier for emotion classification and many of them obtained a low recognition accuracy among the emotions. Most of the accuracies are not higher than 80%. There are different types of kernel functions for the SVM algorithm that can be used to perform the classification tasks such as linear, non-linear, RBF, polynomial, Gaussian, and sigmoid. However, some of the works only mentioned that SVM is their classifier but do not specify what types of kernel they are using. There are also studies that used a neural network as their machine learning algorithm. Most of the authors are using ANN to detect emotions. There are many types of ANN such as deep multilayer perceptron (MLP) [103], recurrent neural network (RNN), long short-term memory (LSTM) [104], and convolutional neural network (CNN) but they do not specify which method of ANN they used in the experiment. As such, more studies need to be conducted to ascertain what actual levels of accuracies can be achieved by the different variants of the classifiers typically used, for example determining what types of SVM kernels or what specific ANN architectures would be able to generate the best classification outcomes.

### 5.5. Multimodal Emotion Detection Using the Combination of Eye-Tracking Data with Other Physiological Signals

Most of the studies used the combination of eye-tracking data with other physiological signals to detect emotions. Many physiological signals can be used to detect emotions such as ECG, EMG, HR, and GSR. However, from the survey, it appears that EEG is most commonly used together with eye-tracking although the accuracy of emotion recognition could still be further enhanced. As such, to improve the performance and achieve higher recognition accuracies, the features of EMG or ECG can be used in combination with eye-tracking data. EMG can be used to record and evaluate the electrical activity produced by the eye muscles. ECG can measure and record the electrical activity of an individual’s heart rhythm. Unimodal emotion recognition usually produces a lower recognition performance. Hence, a multimodal approach of combining of eye-tracking data with other physiological signals will likely enhance the performance of emotion recognition.

### 5.6. Subjects Used in the Experiment

Although most of the studies have used a good balance of male and female subjects in their experiments, the number of subjects used is very often much less than the 30 required to ensure statistical significance. Some of the studies used only five subjects and often less than 10 subjects. Due to the limited number of subjects, the performance and result obtained may not be generalizable. To obtain fiable results, future researchers in this domain should target to use at least 30 subjects in their experiments.

### 5.7. Significant Difference of Accuracy Between Emotion Classes

From the survey, it is quite apparent that there is a big difference between the classification accuracies for different types of emotions. The happy or positive emotions generally tend to have a higher accuracy compared to negative emotions and neutral emotions. Future research work should look into recognizing these more challenging classes of emotions, particularly such as those with negative valence and low arousal responses.

### 5.8. Inter-Subject and Intra-Subject Variability

Most of the studies reviewed in this survey presented their obtained results without clearly discussing and comparing between inter-subject and intra-subject classification accuracy rates. This is in fact a very important criterion in assessing the usefulness of the emotion recognition outcomes. A very high recognition rate may actually be applicable only to intra-subject classification, which would mean that the proposed approach would need a complete retraining cycle before the approach could be used on a new user or test subject. On the other hand, if good emotion recognition results were obtained for inter-subject classification, this would then mean that the solution is ready to be deployed for any future untested user since it is able to work across different users with a high classification accuracy without having to retrain the classification system.

### 5.9. Devices and Applications

More research should also be conducted on how other more readily available eye-tracking approaches can be deployed, such as using the camera found on smartphones. The ability to harness the ubiquity and prevalence of smartphones among everyday users would tremendously expand the scope of possible deployment and practical usage to the everyday consumer. It has recently been shown that extraction of relevant eye-tracking features could be accomplished using convolutional neural networks from images captured from a smartphone camera [105]. Moreover, other possible applications from using eye-tracking and emotion recognition could vastly expand the applicability of such an approach. For example, eye-tracking in the form of gaze concentration has been studied for meditation purposes [106], and the further research of how the performance of such a system could be improved through augmentation of emotion recognition would be highly beneficial since the ability to engage in meditative states has become popular in modern society. Another potentially useful area to investigate for integration would be in advanced driving assistance systems (ADAS), such as in driverless vehicles. Both emotion recognition and eye-tracking have been investigated in ADAS [107] but as separate systems, hence integrating both approaches would likely be beneficial. Another potentially useful area to investigate would be in smart home applications. Similar to ADAS, emotion recognition and eye-tracking have respectively been studied for smart home integration [108]. A smart home that is able to detect an occupant’s emotion via eye-tracking would enable advanced applications such as adjusting the mood and ambient surroundings to best suit the occupant’s current state of mind, such as the ability to detect an occupant is who is feeling stressed and adjusting the lighting or music system to calm the occupant’s emotions.

## 6. Conclusions

In this paper, we have attempted to review eye-tracking approaches for the task of emotion recognition. It was found that there is in fact only a limited number of papers that have been published on using eye-tracking for emotion recognition. Typically, eye-tracking methods were combined with EEG, and as such there is no substantial conclusion yet as to whether eye-tracking alone could be used reliably for emotion recognition. We have also presented a summary of the reviewed papers on emotion recognition with regards to the emotional-relevant features obtainable from eye-tracking data such as pupil diameter, EOG, pupil position, fixation duration of the eye, distance between sclera and iris, motion speed of the eye, and pupillary responses. Some challenges and problems also are presented in this paper for further research. We hope that this survey can assist future researchers who are interested to attempt to conduct research on emotion recognition using eye-tracking technologies to rapidly navigate the published literature in this research domain. 

## References

[1] Verschuere B., Crombez G., Koster E.H., Uzieblo K. (2006). Psychopathy and Physiological Detection of Concealed Information: A review. Psychol. Belg..

[2] Card S.K., Moran T.P., Newell A. (1980). The keystroke-level model for user performance time with interactive systems. Commun. ACM.

[3] Fischer G. (2001). User Modeling in Human–Computer Interaction. User Model. User-Adapt. Interact..

[4] Cowie R., Douglas-Cowie E., Tsapatsoulis N., Votsis G., Kollias S., Fellenz W., Taylor J. (2001). Emotion recognition in human-computer interaction. IEEE Signal Process. Mag..

[5] Zhang Y.-D., Yang Z.-J., Lu H., Zhou X.-X., Phillips P., Liu Q.-M., Wang S. (2016). Facial Emotion Recognition based on Biorthogonal Wavelet Entropy, Fuzzy Support Vector Machine, and Stratified Cross Validation. IEEE Access.

[6] Shu L., Xie J., Yang M., Li Z., Li Z., Liao D., Xu X., Yang X. (2018). A Review of Emotion Recognition Using Physiological Signals. Sensors.

[7] Hess E.H., Polt J.M., Suryaraman M.G., Walton H.F. (1960). Pupil Size as Related to Interest Value of Visual Stimuli. Science.

[8] Rayner K. (2009). The 35th Sir Frederick Bartlett Lecture: Eye movements and attention in reading, scene perception, and visual search. Q. J. Exp. Psychol..

[9] Lohse G.L., Johnson E. (1996). A Comparison of Two Process Tracing Methods for Choice Tasks. Organ. Behav. Hum. Decis. Process..

[10] Bulling A., A Ward J., Gellersen H., Tröster G. (2011). Eye Movement Analysis for Activity Recognition Using Electrooculography. IEEE Trans. Pattern Anal. Mach. Intell..

[11] Cabanac M. (2002). What is emotion?. Behav. Process..

[12] Daniel L. (2011). Psychology.

[13] Mauss I.B., Robinson M.D. (2009). Measures of emotion: A review. Cogn. Emot..

[14] Scherer K.R. (2005). What are emotions? And how can they be measured?. Soc. Sci. Inf..

[15] Colombetti G. (2009). From affect programs to dynamical discrete emotions. Philos. Psychol..

[16] Ekman P. (2005). Basic Emotions. Handb. Cogn. Emot..

[17] Plutchik R. (2002). Nature of emotions. Am. Sci..

[18] Jabreel M., Moreno A. (2019). A Deep Learning-Based Approach for Multi-Label Emotion Classification in Tweets. Appl. Sci..

[19] Russell J. (1980). A circumplex model of affect. J. Personality Soc. Psychol..

[20] Rubin D.C., Talarico J.M. (2009). A comparison of dimensional models of emotion: Evidence from emotions, prototypical events, autobiographical memories, and words. Memory.

[21] Soleymani M., Pantic M., Pun T. (2011). Multimodal Emotion Recognition in Response to Videos. IEEE Trans. Affect. Comput..

[22] Choi K.-H., Kim J., Kwon O.S., Kim M.J., Ryu Y., Park J.-E. (2017). Is heart rate variability (HRV) an adequate tool for evaluating human emotions? – A focus on the use of the International Affective Picture System (IAPS). Psychiatry Res. Neuroimaging.

[23] Lang P.J. (2005). International Affective Picture System (IAPS): Affective Ratings of Pictures and Instruction Manual.

[24] Jacob R.J., Karn K.S. (2003). Eye Tracking in Human-Computer Interaction and Usability Research. The Mind’s Eye.

[25] Singh H., Singh J. (2012). Human eye-tracking and related issues: A review. Int. J. Sci. Res. Publ..

[26] Alghowinem S., AlShehri M., Goecke R., Wagner M. (2014). Exploring Eye Activity as an Indication of Emotional States Using an Eye-Tracking Sensor. Advanced Computational Intelligence in Healthcare-7.

[27] Hess E.H. (1995). The Tell-Tale Eye: How Your Eyes Reveal Hidden thoughts and Emotions.

[28] Isaacowitz D.M., Wadlinger H.A., Goren D., Wilson H.R. (2006). Selective preference in visual fixation away from negative images in old age? An eye-tracking study. Psychol. Aging.

[29] (2018). Looxid Labs, “What Happens When Artificial Intelligence Can Read Our Emotion in Virtual Reality,” Becoming Human: Artificial Intelligence Magazine. https://becominghuman.ai/what-happens-when-artificial-intelligence-can-read-our-emotion-in-virtual-reality-305d5a0f5500.

[30] Mala S., Latha K. (2014). Feature Selection in Classification of Eye Movements Using Electrooculography for Activity Recognition. Comput. Math. Methods Med..

[31] Lu Y., Zheng W.L., Li B., Lu B.L. Combining eye movements and EEG to enhance emotion recognition. Proceedings of the Twenty-Fourth International Joint Conference on Artificial Intelligence.

[32] Lin Y.P., Wang C.H., Jung T.P., Wu T.L., Jeng S.K., Duann J.R., Chen J.H. (2010). EEG-based emotion recognition in music listening. IEEE Trans. Biomed. Eng..

[33] Partala T., Surakka V. (2003). Pupil size variation as an indication of affective processing. Int. J. Hum. -Comput. Stud..

[34] Bradley M., Lang P.J. (1999). The International Affective Digitized Sounds (IADS): Stimuli, Instruction Manual and Affective Ratings.

[35] Cohen J., MacWhinney B., Flatt M., Provost J. (1993). PsyScope: An interactive graphic system for designing and controlling experiments in the psychology laboratory using Macintosh computers. Behav. Res. Methods Instrum. Comput..

[36] Oliva M., Anikin A. (2018). Pupil dilation reflects the time course of emotion recognition in human vocalizations. Sci. Rep..

[37] Gilzenrat M.S., Nieuwenhuis S., Jepma M., Cohen J.D. (2010). Pupil diameter tracks changes in control state predicted by the adaptive gain theory of locus coeruleus function. Cogn. Affect. Behav. Neurosci..

[38] Peirce J.W. (2007). PsychoPy—Psychophysics software in Python. J. Neurosci. Methods.

[39] Belin P., Fillion-Bilodeau S., Gosselin F. (2008). The Montreal Affective Voices: A validated set of nonverbal affect bursts for research on auditory affective processing. Behav. Res. Methods.

[40] Hastie T., Tibshirani R. (1990). Generalized Additive Models.

[41] Mehler M.F., Purpura M.P. (2008). Autism, fever, epigenetics and the locus coeruleus. Brain Res. Rev..

[42] Zheng W.-L., Dong B.-N., Lu B.-L. (2014). Multimodal emotion recognition using EEG and eye-tracking data. Proceedings of the 2014 36th Annual International Conference of the IEEE Engineering in Medicine and Biology Society.

[43] Lanatà A., Armato A., Valenza G., Scilingo E.P. (2011). Eye tracking and pupil size variation as response to affective stimuli: A preliminary study. Proceedings of the 5th International ICST Conference on Pervasive Computing Technologies for Healthcare.

[44] Schreiber K.M., Haslwanter T. (2004). Improving Calibration of 3-D Video Oculography Systems. IEEE Trans. Biomed. Eng..

[45] Chen W., Er M.J., Wu S. (2006). Illumination compensation and normalization for robust face recognition using discrete cosine transform in logarithm domain. IEEE Trans. Syst. ManCybern. Part B (Cybern).

[46] Land E.H., McCann J.J. (1971). Lightness and Retinex Theory. J. Opt. Soc. Am..

[47] Sheer P. (1997). A software Assistant for Manual Stereo Photometrology. Ph.D. Thesis.

[48] Cover T., Hart P. (1967). Nearest neighbor pattern classification. IEEE Trans. Inf. Theory.

[49] Stehman S.V. (1997). Selecting and interpreting measures of thematic classification accuracy. Remote. Sens. Environ..

[50] Wong B.S.F., Ho G.T.S., Tsui E. (2017). Development of an intelligent e-healthcare system for the domestic care industry. Ind. Manag. Data Syst..

[51] Sodhro A.H., Sangaiah A.K., Sodhro G.H., Lohano S., Pirbhulal S. (2018). An Energy-Efficient Algorithm for Wearable Electrocardiogram Signal Processing in Ubiquitous Healthcare Applications. Sensors.

[52] Begum S., Barua S., Ahmed M.U. (2014). Physiological Sensor Signals Classification for Healthcare Using Sensor Data Fusion and Case-Based Reasoning. Sensors.

[53] Wang Y., Lv Z., Zheng Y. (2018). Automatic Emotion Perception Using Eye Movement Information for E-Healthcare Systems. Sensors.

[54] Paul S., Banerjee A., Tibarewala D.N. (2017). Emotional eye movement analysis using electrooculography signal. Int. J. Biomed. Eng. Technol..

[55] Primer A., Burrus C.S., Gopinath R.A. (1998). Introduction to Wavelets and Wavelet Transforms.

[56] Hjorth B. (1970). EEG analysis based on time domain properties. Electroencephalogr. Clin. Neurophysiol..

[57] Aracena C., Basterrech S., Snael V., Velasquez J., Claudio A., Sebastian B., Vaclav S., Juan V. (2015). Neural Networks for Emotion Recognition Based on Eye Tracking Data. Proceedings of the 2015 IEEE International Conference on Systems, Man, and Cybernetics.

[58] Jänig W. (1985). The Autonomic Nervous System. Fundamentals of Neurophysiology.

[59] Cheng B., Titterington D.M. (1994). Neural Networks: A Review from a Statistical Perspective. Stat. Sci..

[60] Palm R.B. (2012). Prediction as a Candidate for Learning Deep Hierarchical Models of Data.

[61] Anwar S.A. (2019). Real Time Facial Expression Recognition and Eye Gaze Estimation System (Doctoral Dissertation).

[62] Cootes T.F., Taylor C., Cooper D., Graham J. (1995). Active Shape Models-Their Training and Application. Comput. Vis. Image Underst..

[63] Edwards G.J., Taylor C., Cootes T.F. (1998). Interpreting face images using active appearance models. Proceedings of the Third IEEE International Conference on Automatic Face and Gesture Recognition.

[64] Gomez-Ibañez A., Urrestarazu E., Viteri C. (2014). Recognition of facial emotions and identity in patients with mesial temporal lobe and idiopathic generalized epilepsy: An eye-tracking study. Seizure.

[65] Meletti S., Benuzzi F., Rubboli G., Cantalupo G., Maserati M.S., Nichelli P., Tassinari C.A. (2003). Impaired facial emotion recognition in early-onset right mesial temporal lobe epilepsy. Neurol..

[66] Circelli K.S., Clark U., Cronin-Golomb A. (2012). Visual scanning patterns and executive function in relation to facial emotion recognition in aging. AgingNeuropsychol. Cogn..

[67] Firestone A., Turk-Browne N.B., Ryan J.D. (2007). Age-Related Deficits in Face Recognition are Related to Underlying Changes in Scanning Behavior. AgingNeuropsychol. Cogn..

[68] Wong B., Cronin-Golomb A., Neargarder S. (2005). Patterns of Visual Scanning as Predictors of Emotion Identification in Normal Aging. Neuropsychol..

[69] Malcolm G.L., Lanyon L., Fugard A., Barton J.J.S. (2008). Scan patterns during the processing of facial expression versus identity: An exploration of task-driven and stimulus-driven effects. J. Vis..

[70] Nusseck M., Cunningham D.W., Wallraven C., De Tuebingen M.A., De Tuebingen D.A.G.-, De Tuebingen C.A., Bülthoff H.H., De Tuebingen H.A. (2008). The contribution of different facial regions to the recognition of conversational expressions. J. Vis..

[71] Ekman P., Friesen W.V. (2003). Unmasking the Face: A Guide to Recognizing Emotions from Facial Clues.

[72] Benton A.L., Abigail B., Sivan A.B., Hamsher K.D., Varney N.R., Spreen O. (1994). Contributions to Neuropsychological Assessment: A clinical Manual.

[73] Tsang V., Tsang K.L.V. (2016). Eye-tracking study on facial emotion recognition tasks in individuals with high-functioning autism spectrum disorders. Autism.

[74] Bal E., Harden E., Lamb D., Van Hecke A.V., Denver J.W., Porges S.W. (2009). Emotion Recognition in Children with Autism Spectrum Disorders: Relations to Eye Gaze and Autonomic State. J. Autism Dev. Disord..

[75] Carl L. (1847). On the influence of respiratory movements on blood flow in the aortic system [in German]. Arch Anat Physiol Leipzig..

[76] Hayano J., Sakakibara Y., Yamada M., Kamiya T., Fujinami T., Yokoyama K., Watanabe Y., Takata K. (1990). Diurnal variations in vagal and sympathetic cardiac control. Am. J. Physiol. Circ. Physiol..

[77] Porges S.W. (1986). Respiratory Sinus Arrhythmia: Physiological Basis, Quantitative Methods, and Clinical Implications. Cardiorespiratory and Cardiosomatic Psychophysiology.

[78] Pagani M., Lombardi F., Guzzetti S., Rimoldi O., Furlan R., Pizzinelli P., Sandrone G., Malfatto G., Dell’Orto S., Piccaluga E. (1986). Power spectral analysis of heart rate and arterial pressure variabilities as a marker of sympatho-vagal interaction in man and conscious dog. Circ. Res..

[79] Porges S.W., Cohn J.F., Bal E., Lamb D. (2007). The Dynamic Affect Recognition Evaluation [Computer Software].

[80] Grossman P., Beek J., Wientjes C. (1990). A Comparison of Three Quantification Methods for Estimation of Respiratory Sinus Arrhythmia. Psychophysiology.

[81] Kamen G. (2004). Electromyographic kinesiology. Research Methods in Biomechanics.

[82] Boraston Z., Blakemore S.J. (2007). The application of eye-tracking technology in the study of autism. J. Physiol..

[83] Pioggia G., Igliozzi R., Ferro M., Ahluwalia A., Muratori F., De Rossi D. (2005). An Android for Enhancing Social Skills and Emotion Recognition in People With Autism. IEEE Trans. Neural Syst. Rehabil. Eng..

[84] Lischke A., Berger C., Prehn K., Heinrichs M., Herpertz S.C., Domes G. (2012). Intranasal oxytocin enhances emotion recognition from dynamic facial expressions and leaves eye-gaze unaffected. Psychoneuroendocrinology.

[85] Heinrichs M., Von Dawans B., Domes G. (2009). Oxytocin, vasopressin, and human social behavior. Front. Neuroendocr..

[86] Rajakumari B., Selvi N.S. (2016). HCI and eye-tracking: Emotion recognition using hidden markov model. Int. J. Comput. Sci. Netw. Secur..

[87] Baum L.E., Petrie T. (1966). Statistical Inference for Probabilistic Functions of Finite State Markov Chains. Ann. Math. Stat..

[88] Baum L.E., Eagon J.A. (1967). An inequality with applications to statistical estimation for probabilistic functions of Markov processes and to a model for ecology. Bull. Am. Math. Soc..

[89] Baum L.E., Sell G. (1968). Growth transformations for functions on manifolds. Pac. J. Math..

[90] Baum L.E., Petrie T., Soules G., Weiss N. (1970). A Maximization Technique Occurring in the Statistical Analysis of Probabilistic Functions of Markov Chains. Ann. Math. Stat..

[91] Baum L.E. (1972). An Inequality and Associated Maximization Technique in Statistical Estimation of Probabilistic Functions of a Markov Process. Inequalities.

[92] Ulutas B.H., Ozkan N., Michalski R. (2019). Application of hidden Markov models to eye tracking data analysis of visual quality inspection operations. Cent. Eur. J. Oper. Res..

[93] Chuk T., Chan A.B., Hsiao J.H. (2014). Understanding eye movements in face recognition using hidden Markov models. J. Vis..

[94] Raudonis V., Dervinis G., Vilkauskas A., Paulauskaite A., Kersulyte G. (2013). Evaluation of Human Emotion from Eye Motions. Int. J. Adv. Comput. Sci. Appl..

[95] McCulloch W.S., Pitts W. (1943). A logical calculus of the ideas immanent in nervous activity. Bull. Math. Boil..

[96] Alhargan A., Cooke N., Binjammaz T. (2017). Affect recognition in an interactive gaming environment using eye tracking. Proceedings of the 2017 Seventh International Conference on Affective Computing and Intelligent Interaction (ACII).

[97] De Melo C.M., Paiva A., Gratch J. (2014). Emotion in Games. Handbook of Digital Games.

[98] Zeng Z., Pantic M., Roisman G., Huang T.S. (2008). A Survey of Affect Recognition Methods: Audio, Visual, and Spontaneous Expressions. IEEE Trans. Pattern Anal. Mach. Intell..

[99] Rani P., Liu C., Sarkar N., Vanman E.J. (2006). An empirical study of machine learning techniques for affect recognition in human–robot interaction. Pattern Anal. Appl..

[100] Purves D. (2009). Neuroscience. Sch..

[101] Alhargan A., Cooke N., Binjammaz T. (2017). Multimodal affect recognition in an interactive gaming environment using eye tracking and speech signals. Proceedings of the 19th ACM International Conference on Multimodal Interaction - ICMI 2017.

[102] Giannakopoulos T. (2009). A Method for Silence Removal and Segmentation of Speech Signals, Implemented in Matlab.

[103] Rosenblatt F. (1961). Principles of Neurodynamics. Perceptrons and the Theory of Brain Mechanisms.

[104] Hochreiter S., Schmidhuber J. (1997). Long short-term memory. Neural Comput..

[105] Brousseau B., Rose J., Eizenman M. (2020). Hybrid Eye-Tracking on a Smartphone with CNN Feature Extraction and an Infrared 3D Model. Sensors.

[106] Chang K.-M., Chueh M.-T.W. (2019). Using Eye Tracking to Assess Gaze Concentration in Meditation. Sensors.

[107] Khan M.Q., Lee S. (2019). Gaze and Eye Tracking: Techniques and Applications in ADAS. Sensors.

[108] Bissoli A., Lavino-Junior D., Sime M., Encarnação L.F., Bastos-Filho T.F. (2019). A Human–Machine Interface Based on Eye Tracking for Controlling and Monitoring a Smart Home Using the Internet of Things. Sensors.

